# Device for easily measuring of the torque of an implant hand driver

**DOI:** 10.1186/s40064-016-3071-y

**Published:** 2016-08-22

**Authors:** Yuji Sato, Tokiko Osawa, Noboru Kitagawa

**Affiliations:** Department of Geriatric Dentistry, School of Dentistry, Showa University, 2-1-1 Kitasenzoku, Ota-ku, Tokyo, 145-8515 Japan

**Keywords:** Torque, Fastening, Load, Appropriate torque value

## Abstract

**Background:**

As one of the loads applied in implant dentistry, managing the torque is important for the success of an implant treatment. For this purpose, it is crucial to ascertain the level of torque being exerted with a hand driver. We have developed an adapter that makes it easy to measure torque by using a standard torque wrench in one’s possession, rather than with a torquemeter.

**Procedures:**

The head of an abutment screw is cut into a hexagon and pushed into and fixed to the hexagonal hole (for multi-unit abutments) of a machine driver. With this, a torque wrench adapter and torque wrench are assembled. A hand driver is rotated clockwise to the limit, and the torque value is read.

**Results:**

It was possible to read the torque value during screw fastening.

**Conclusions:**

This technique makes it easy for each dentist to measure the maximum torque that can be exerted by a hand driver he or she is using. It is even possible to handle different implant systems.

## Background

Fastening screws with the appropriate torque is very important for the success of an implant. First, fastening is done with a hand driver, and then final fastening must be done at a certain torque with a torque wrench. It is very important that to know whether we can exert a torque of how much with hand driver in advance. However, dentists may not have the same torque wrench that is used in the implant system of another dental clinic. There are also implant systems that do not have a torque wrench, and it is important for each dentist to know what torque can be exerted with the hand driver. Doing so requires the use of a torquemeter (Shiigai 2007), but it is not realistic for a general-practice dentist to be equipped with a torquemeter. Therefore, we have developed and introduce here a simple adapter that makes it possible to use a standard torque wrench already in one’s possession to measure the torque that can be exerted with a hand driver.

## Procedures

Nobel Biocare’s torque wrench (Brånemark System^®^ Torque wrench, Nobel Biocare, Göteborg, Sweden) is described by way of example.

1. Prepare a machine driver (for multi-unit abutments) (hexagonal hole with a major axis of 2.5 mm) (Brånemark System^®^ Screwdriver Machine Multi-unit, Nobel Biocare, Göteborg, Sweden) that can be used with a torque wrench, and an abutment screw (head with a diameter of 2.5 mm) (Brånemark System^®^ abutment screw, Nobel Biocare, Göteborg,Sweden) (Fig. 1).

**Fig. 1:**
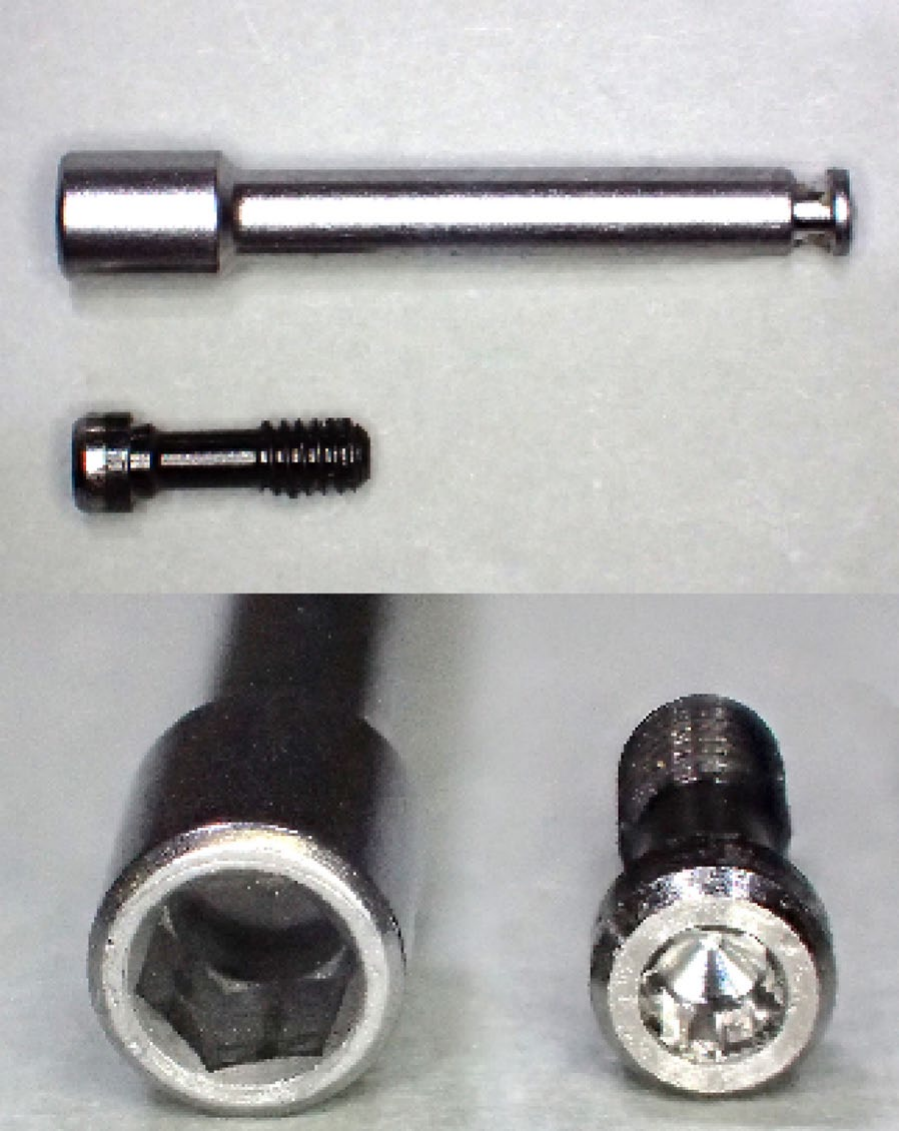
Machine driver (for multi-unit abutments) and abutment screw
2. Under a dental microscope, cut the head of the abutment screw into a hexagonal shape (Fig. 2). Confirm entry of the head into the hexagonal hole of the driver.

**Fig. 2:**
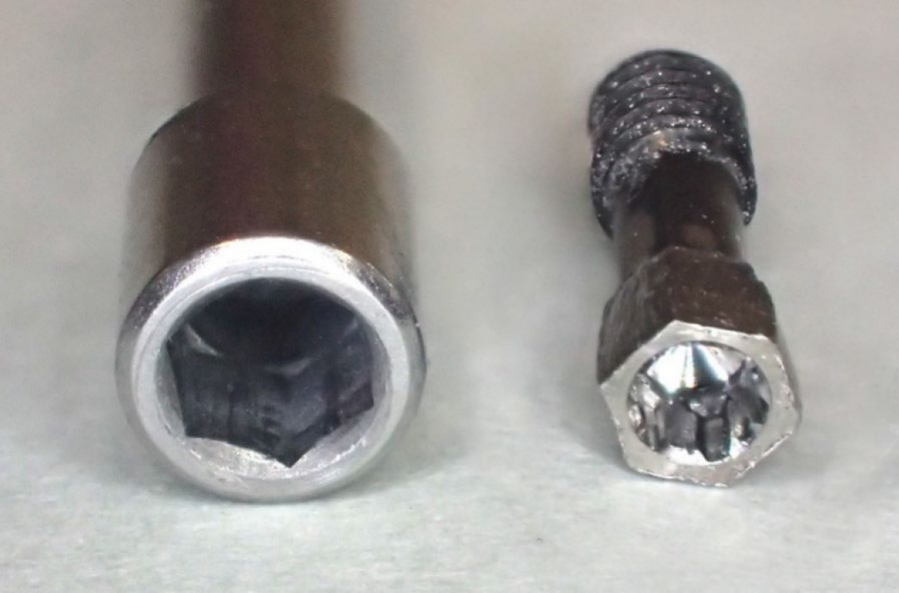
Abutment screw with head cut into a hexagonal shape
3. Remove the screw part of the abutment screw, and push into the hexagonal hole of the driver (Fig. 3). If it is loose, apply instant adhesive and then push it in.

**Fig. 3:**
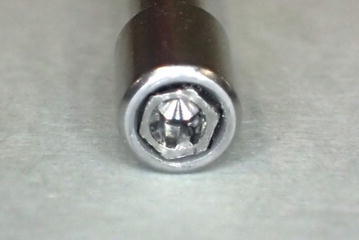
Abutment screw mounted onto a driver
4. Prepare the torque wrench, hand driver, machined abutment driver, and torque wrench adapter (Fig. 4) and assemble.

**Fig. 4:**
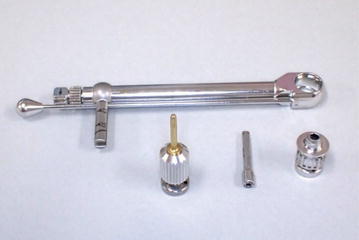
Preparation of a torque wrench, hand driver, machined abutment driver, and torque wrench adapter
5. Holding down the lever with the left hand, rotate the hand driver clockwise (when fastening the screw) to the limit with the right hand, and read the torque value (Fig. 5).

**Fig. 5:**
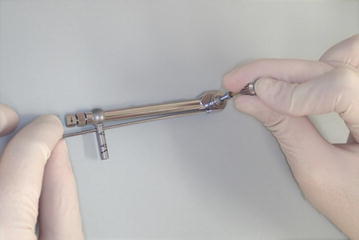
Reading of the torque value exerted with the hand driver
6. Reassemble the torque wrench, and measure the counterclockwise (when loosening the screw) torque.

## Discussion

We have introduced a simple technique for mounting a hand driver onto a torque wrench. This technique makes it easy for each dentist to measure the maximum torque that can be exerted by a hand driver he or she is using. Even when using different implant systems, similar machining can be done to make components that can be mounted onto a torque wrench.

The machined driver can be mounted onto a handpiece for implant placement that indicates the torque; with a combined hand driver, the torque can also be indicated as a numeric value.

## Conclusions

We have introduced a method by which machining a commercially-available machine driver and screw makes it possible to mount a hand driver onto a torque wrench, making it easy for dentists to measure the torque that can be exerted with the hand drivers they are using.
